# Data on the effect of knockout of neruregulin-1 type III on Remak bundle structure

**DOI:** 10.1016/j.dib.2018.03.099

**Published:** 2018-03-27

**Authors:** Yuki Miyamoto, Tomohiro Torii, Masashi Inoue, Takako Morimoto, Masahiro Yamamoto, Junji Yamauchi

**Affiliations:** a Laboratory of Molecular Neuroscience and Neurology, School of Life Sciences, Tokyo University of Pharmacy and Life Sciences, Hachioji, Tokyo 192-0355, Japan; b Department of Pharmacology, National Research Institute for Child Health and Development, Setagaya, Tokyo 157-8535, Japan; c Department of Neuroscience, Baylor College of Medicine, Houston, TX 77030, USA; d Tsumura Research Laboratories, Tsumura & Co., Inashiki, Ibaraki 200-1192, Japan

**Keywords:** Neuregulin-1, Knockout, Remak bundle, Sciatic nerve

## Abstract

Schwann cells in the peripheral nervous system wrap around large diameter axons to form the myelin sheath, that contains one axon. Schwann cells also wrap around small diameter axons to form the Remak bundle, that contains many axons. Neuregulin-1 (NRG1) type III binds Schwann cell plasma membrane ErbB2/3 receptor to regulate morphological changes of Schwann cells. Herein we provide the data on the effect of NRG1 type III knockout (Miyamoto et al., 2017) [1] on the Remak bundle structure. Since complete knockout mice of NRG1type III are embryonically lethal, we have usedNRG1type III (+/−) mice's sciatic nerves in these experiments.

**Specifications table**

**Table:** 

Subject area	Biology
More specific subject area	Molecular and cellular neuroscience, Neurobiology
Type of data	Figure, Graph
How data was acquired	Electron microscopy, immunoblotting
Data format	Raw and analyzed data
Experimental factors	NRG1 knockout mice were used for experiments
Experimental features	Electron microscopic and immunoblotting analyses
Data source location	Laboratory of Molecular Neuroscience and Neurology, School of Life Sciences, Tokyo University of Pharmacy and Life Sciences, Tokyo, Japan
Data accessibility	Data is available with this article

**Value of the data**

• This data set is of value to the scientific community to need the information for the biological effect of a growth factor, especially one in the nervous system.
• The data allow us to promote our understanding of how a growth factor plays a role in forming the peripheral nervous system.

## 1 Data

**Fig. 1:**
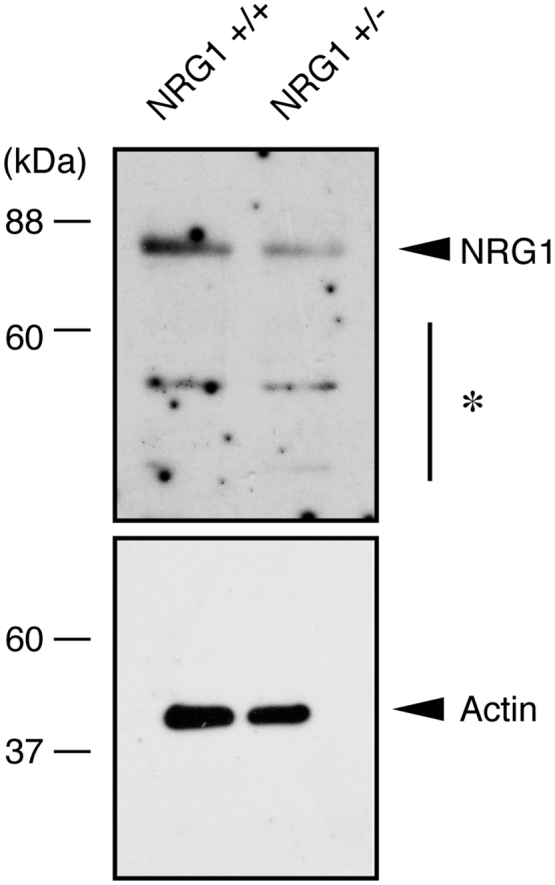
Immunoblotting images of NRG1 type III knockout mice (+/−) and littermate controls (+/+). Sciatic nerve tissue lysates from NRG1 type III knockout mice and littermate controls were immunoblotted with an antibody against NRG1 (60–88 kDa) or control actin (~40 kDa). Asterisk indicates probable NRG1 degradation products.

**Fig. 2:**
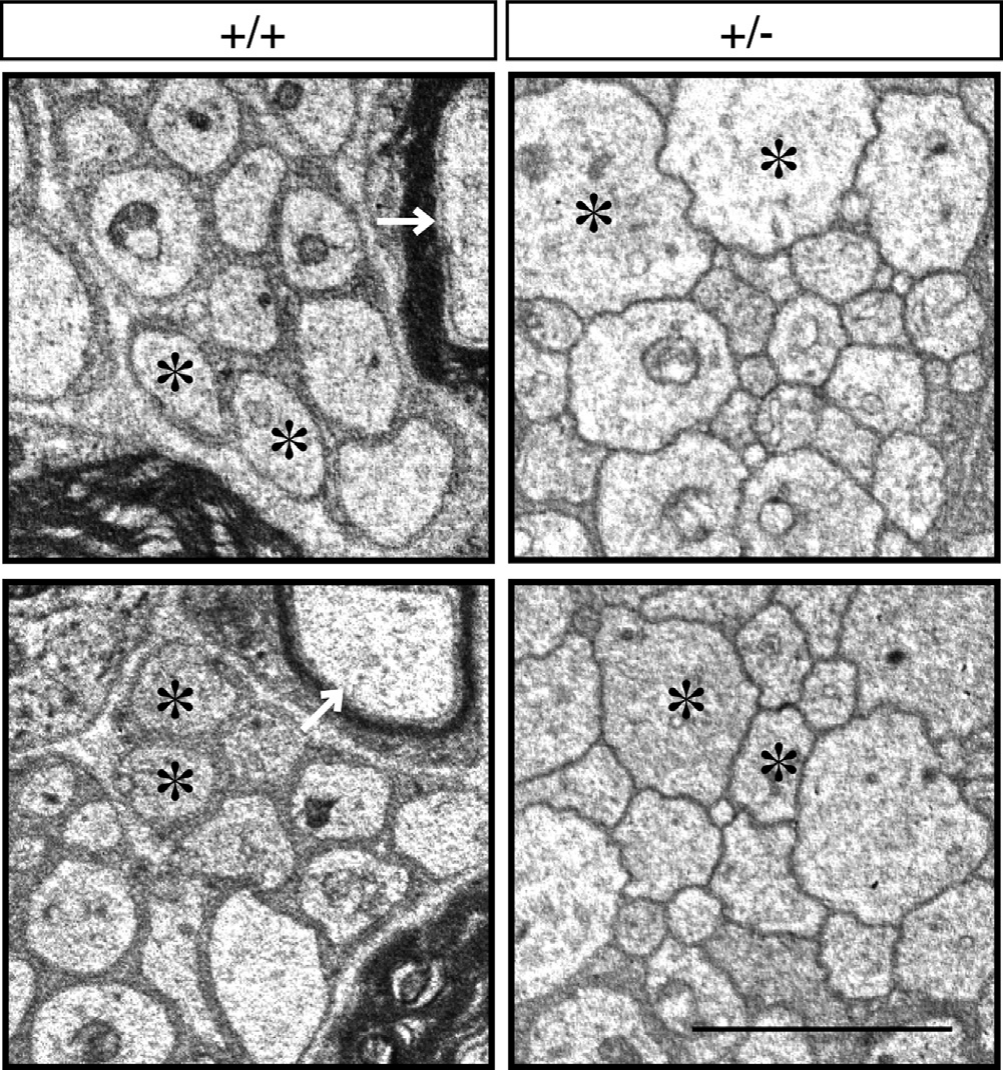
Electron microscopic images of NRG1 type III +/-and +/+ mouse Remak bundles. Representative electron microscopic images (2500-fold) of cross sections in 2-month-old NRG1 type III knockout mice and littermate controls are shown. Asterisks indicate small diameter axons in Remak bundles. Arrows indicate large diameter axons surrounded with myelin sheaths. Scale bar shows1 μm.

**Fig. 3:**
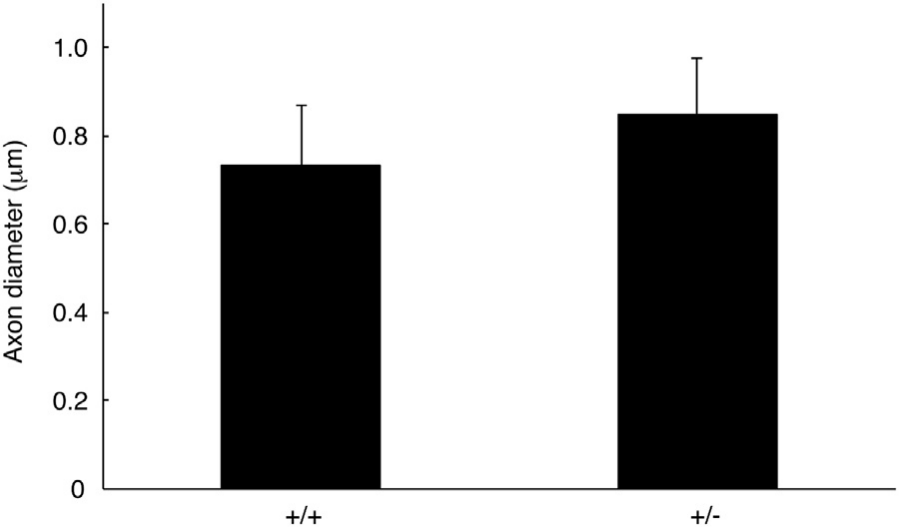
Diameters of axon in Remak bundles of NRG1 type III +/− and +/+ mice. The length of axon diameters in Remak bundles of 2-month-old NRG1 type III knockout mice and littermate controls was measured (the *p* value=0.191 [not significant], *n*=6; unpaired Student's *t*-test).

**Fig. 4:**
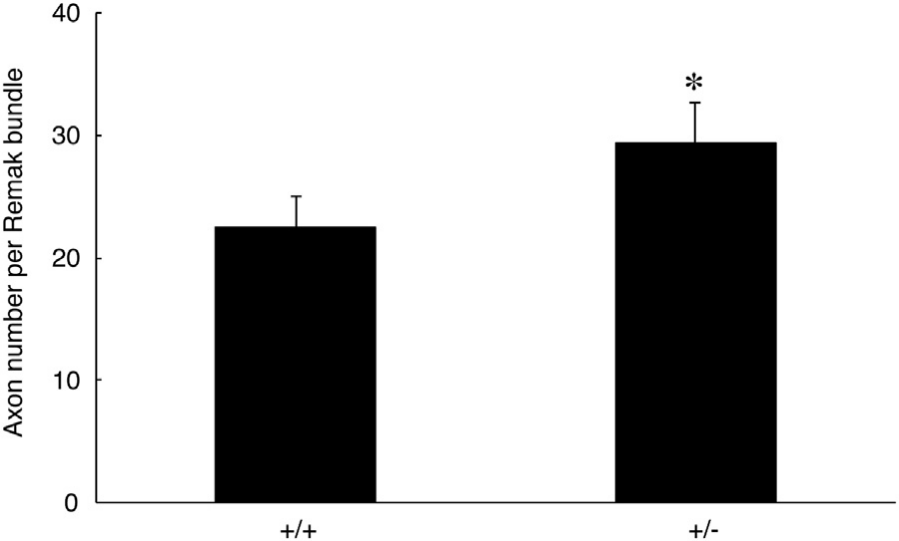
Number of axons in Remak bundles of NRG1 type III +/− and +/+ mice. The number of axons in Remak bundles of 2-month-old NRG1type III knockout mice and littermate controls was measured (*, the *p* value=0.00442, *n*=6; unpaired Student's *t*-test).

**Fig. 5:**
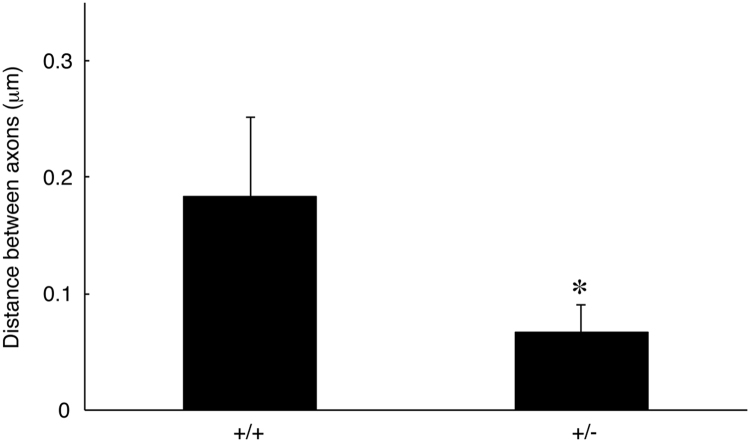
Distance between axons of NRG1 type III +/− and +/+ mice. The distance between an axon and a neighboring axon in Remak bundles of 2-month-old NRG1 type III knockout mice and littermate controls was measured (*, the *p* value=0.00492, *n*=6; unpaired Student's *t*-test).

The data shared in this article provide electron microscopic analyses of Remak bundles in the peripheral nervous system [2], [3]. Schwann cells surround the axons with less than 1 μm of diameters to form Remak bundle. Immunoblotting confirmed that NRG1 type III knockout mice (+/−) [1] exhibit less expression of NRG1 type III in sciatic nerves (Fig. 1). The length of axon diameters in Remak bundles was comparable in NRG1 type III knockout mice and littermate controls (Fig. 2, Fig. 3). On the other hand, NRG1 type III knockout mice exhibited more numbers of axons in Remak bundles than the controls (Fig. 2, Fig. 4). Also, knockout mice exhibited short distance between an axon and a neighboring axon, comparing with the controls (Fig. 2, Fig. 5).

## 2 Experimental design, materials and methods

### 2.1 Electron microscopy

Sciatic nerves were fixed with 2% paraformaldehyde and 2% glutaraldehyde in 0.1% cacodylate buffer. The tissues were post fixed with buffered 2% osmium tetroxide, dehydrated with an ethanol gradient, treated with acetone, and embedded in epoxy resin. Ultrathin sections of cross sections were stained with uranyl acetate and lead citrate. They were observed and photographed with Hitachi electron microscopes [1], [4].

### 2.2 Immunoblotting

Tissues were lysed in lysis buffer A (50 mM HEPES-NaOH, pH 7.5, 20 mM MgCl_2_, 150 mM NaCl, 1 mM dithiothreitol, 1 mM phenylmethane sulfonylfluoride, 1 μg/ml leupeptin, 1 mM EDTA, 1 mM Na_3_VO_4_, and 10 mM NaF) containing biochemical detergents (0.5% NP-40, 1% CHAPS, and 0.3% SDS). Unless otherwise indicated, all lysis steps were performed at 4 °C [4], [5]. The proteins in the cell supernatants were denatured, subjected to SDS-PAGE, and blotted to a PVDF membrane using the TransBlot TurboTransfer System (Bio-Rad). The membranes were blocked with a Blocking One reagent (Nacalai), and immunoblotted using primary antibodies (anti-NRG1 type III [intracellular domain] from Santa-Cruz and anti-actin from MBL) followed by peroxidase-conjugated secondary antibodies (GE Healthcare). The bound antibodies were detected using a Chemiluminescence One reagent (Nacalai) and the C-DiGitscanner (MS Systems).
